# Clinicopathological and survival outcomes of 4L lymph node dissection in left lung adenocarcinoma and squamous cell carcinoma

**DOI:** 10.3389/fonc.2023.1124014

**Published:** 2023-04-11

**Authors:** Leilei Shen, Juntang Guo, Weidong Zhang, Chaoyang Liang, Han Chen, Yang Liu

**Affiliations:** ^1^ Postgraduate School, Medical School of Chinese People's Liberation Army (PLA), Beijing, China; ^2^ Department of Thoracic Surgery, Chinese PLA General Hospital, Beijing, China; ^3^ Department of Thoracic Surgery, Hainan Hospital of PLA General Hospital, Sanya, China; ^4^ Department of Information, Hainan Hospital of PLA General Hospital, Sanya, China

**Keywords:** adenocarcinoma, squamous cell carcinoma, station 4L metastasis, prognosis, lymph node dissection

## Abstract

**Background:**

Whether 4L lymph node dissection (LND) should be performed remains unclear and controversial. Prior studies have found that station 4L metastasis was not rare and that 4L LND may provide survival benefits. The objective of this study was to analyze the clinicopathological and survival outcomes of 4L LND from the perspective of histology.

**Methods:**

This retrospective study included 74 patients with squamous cell carcinoma (SCC) and 84 patients diagnosed with lung adenocarcinoma (ADC) between January 2008 and October 2020. All patients underwent pulmonary resection with station 4L LND and were staged as T1-4N0-2M0. Clinicopathological features and survival outcomes were investigated based on histology. The study endpoints were disease-free survival (DFS) and overall survival (OS).

**Results:**

The incidence rate of station 4L metastasis was 17.1% (27/158) in the entire cohort, with 8.1% in the SCC group, and 25.0% in the ADC group. No statistical differences in the 5-year DFS rates (67.1% *vs*. 61.7%, *P*=0.812) and 5-year OS rates (68.6% *vs*. 59.3%, *P*=0.100) were observed between the ADC group and the SCC group. Multivariate logistic analysis revealed that histology (SCC *vs*. ADC: OR, 0.185; 95% CI, 0.049–0.706; *P*=0.013) was independently associated with 4L metastasis. Multivariate survival analysis showed that the status of 4L metastasis was an independent factor for DFS (HR, 2.563; 95% CI, 1.282–5.123; *P*=0.008) but not for OS (HR, 1.597; 95% CI, 0.749–3.402; *P*=0.225).

**Conclusion:**

Station 4L metastasis is not rare in left lung cancer. Patients with ADC have a greater predilection for station 4L metastasis and may benefit more from performing 4L LND.

## 1 Introduction

Lung cancer is the leading cause of cancer-related mortality worldwide, with non-small-cell lung cancer (NSCLC) being the most frequent subtype (1). Lobectomy with systemic nodal dissection (SND) remains the fundamental approach for operable NSCLC patients because of its vital role in accurate staging and its therapeutic and prognostic implications (2). The National Comprehensive Cancer Network (NCCN) guidelines require the evaluation of a minimum of three N2 stations. For left-sided cancers, stations 4L, 5, 6, 7, 8, and 9 should be dissected (3). Often, station 4L is not routinely sampled or dissected during lung cancer resection out of concern for recurrent laryngeal nerve, thoracic duct, and aortic arch injury because of its anatomic location (4). Previous studies have shown that station 4L lymph nodes play a crucial role in the left bronchial-recurrent lymph node (LN) chain, an essential lymphatic pathway of the left lung (5, 6). Recently, some studies have assessed the clinical significance of 4L lymph node dissection (4L LND) in left lung cancer and found that station 4L metastasis was not rare and that 4L LND may provide survival benefits (7–12). However, whether 4L LND should be resected remains unclear and controversial because most guidelines have no detailed requirements. Furthermore, complete 4L LND is technically challenging and may cause postoperative complications. Thus, many surgeons choose not to perform 4L LND even in high-volume thoracic centers.

To the best of our knowledge, no study has been conducted to identify the clinicopathological features and survival outcomes of 4L LND in left lung cancer from a histological perspective. Therefore, the purpose of this study was to investigate the differences in clinicopathological features and survival outcomes between adenocarcinoma (ADC) and squamous cell carcinoma (SCC) after 4L LND.

## 2 Patients and methods

### 2.1 Study population

**Figure 1:**
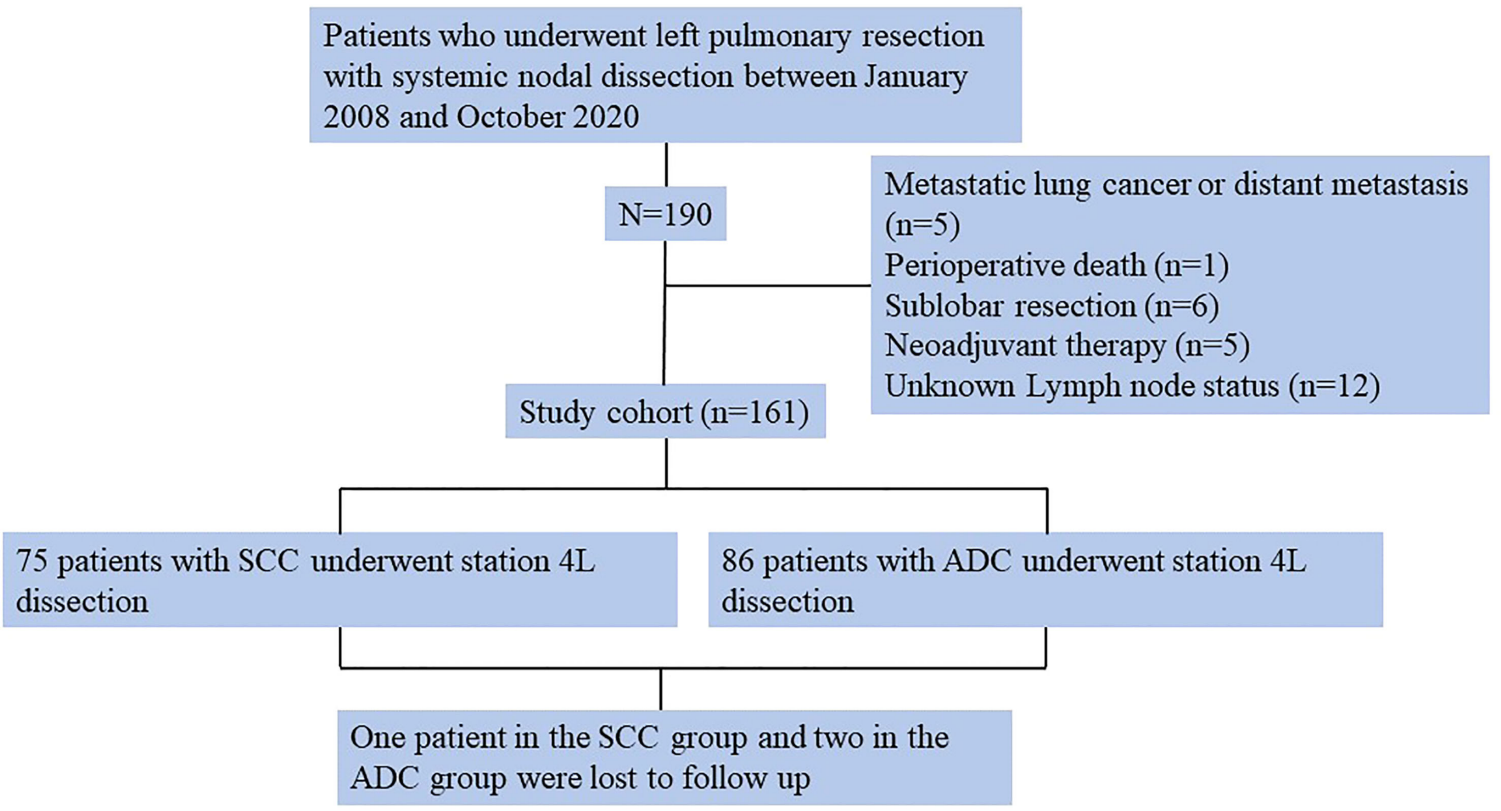
Study enrollment flow diagram.

We retrospectively reviewed the records of all patients who underwent left NSCLC surgery at our hospital between January 2008 and October 2020. The inclusion criteria were as follows: patients who underwent pulmonary resection (lobectomy or pneumonectomy) with SND or lymph node sampling, tumor pathology of ADC or SCC, and pathological stage of T1-4N0-2M0. The following patients were excluded: those with metastatic lung tumors or distant metastasis, those who underwent partial resection or segmentectomy, those who had no LN resection or unknown lymph node status (pNx), and those who received neoadjuvant therapy. Finally, 158 patients were enrolled in this study, and their clinicopathological characteristics were collected from the hospital electronic medical record system (**Figure 1**). All patients were staged according to the 8^th^ edition of the American Joint Committee on Cancer (AJCC) lung cancer staging system (13). The study was approved by the Ethics Committee of Chinese People’s Liberation Army General Hospital. All patients signed the informed consent.

### 2.2 Follow-up

Routine examinations after surgery were requested every 3 months for the first 2 years, and every 6 months thereafter for 5 years. After 5 years, the patients were assessed annually. The examinations included blood tumor marker testing, chest CT, ultrasound of the neck and abdomen, and head magnetic resonance imaging. Bone scans were performed in case of bone pain. Follow-up information was collected by official contact with patients or their relatives *via* telephone or from the hospital outpatient clinic records. The last follow-up was in May 2022. The primary endpoint was disease-free survival (DFS), which was defined as the time interval from the date of surgery to the first event (recurrence, metastasis, or NSCLC-related death) or last follow-up. Overall survival (OS) served as the secondary endpoint, defined as the time interval between the date of surgery and the date of death or the last follow-up. The DFS and OS were calculated in months.

### 2.3 Statistical analysis

All statistical analyses were performed using SPSS version 22 software (IBM Corporation, Armonk, NY, USA). Continuous variables are expressed as the mean with standard deviation as well as the median with a range of values. We used the Mann-Whitney U test to determine significant differences in continuous variables between the two groups. Every group with categorical variables is summarized with the frequency and percentage of the considered population, and statistical comparisons between the two groups were performed using the χ^2^ test. Survival curves were estimated using the Kaplan-Meier method, and the log-rank test was used to compare differences. The Cox proportional hazards model was applied in univariate analyses to determine the influence on patients’ risk of death. Predictive variables were selected based on univariate models (*P*-value <0.05). A two-sided *P*<0.05 was considered statistically significant.

## 3 Results

### 3.1 Patients’ clinical characteristics

**Table 1** shows the clinical characteristics of the patients (n=158). A total of 74 (46.8%) and 84 (53.2%) patients were assigned to the SCC group and ADC group, respectively. Patients in the SCC group were older than those in the ADC group (*P*=0.001). Sixty-seven (90.5%) patients in the SCC group were male, whereas 38 (45.2%) male patients in the ADC group (*P*<0.001). The proportion of smoking history in the SCC group was significantly higher than that in the ADC group (*P*<0.001). Chest CT showed a central mass in 40 (54.1%) SCC cases and in 14 (16/7%) ADC patients (*P*<0.001). No differences were observed in terms of comorbidities, abnormality of tumor markers, tumor location, surgical procedure, surgical approach, lymph node dissection, postoperative complication, and adjuvant therapy.

**Table 1:** Clinical characteristics of patients in the squamous cell carcinoma and adenocarcinoma groups.

Variables	SCC	ADC	*P*
	N=74	N=84	
Age (years)	61.73 ± 9.51	57.55 ± 8.87	0.001
Sex			<0.001
Male	67(90.5%)	38(45.2%)	
Female	7(9.5%)	46(54.8%)	
Smoking history	56(75.7%)	38(45.2%)	<0.001
Comorbidities ^†^	25(33.8%)	28(33.3%)	0.952
Abnormality of tumor markers ^‡^	8(10.8%)	17(20.2%)	0.105
Tumor location			0.348
Left upper lobe	44(59.5%)	56(66.7%)	
Left lower lobe	30(40.5%)	28(33.3%)	
Tumor area			<0.001
Central	40(54.1%)	14(16.7%)	
Peripheral	34(45.9%)	70(83.3%)	
Surgical procedure			0.809
Pneumonectomy	19(25.7%)	23(27.4%)	
Lobectomy	55(74.3%)	61(72.6%)	
Surgical approach			0.083
VATS	26(35.1%)	41(48.8%)	
Thoracotomy	48(64.9%)	43(51.2%)	
Lymph node dissection			
Stations	6.01 ± 0.94	6.23 ± 1.02	0.204
Numbers	16.41 ± 7.88	15.21 ± 7.22	0.371
Postoperative complications			
Hoarseness	5(6.8%)	4(4.8%)	0.735
Chylothorax	0	0	1.000
Adjuvant therapy	41(55.4%)	46(54.8%)	0.935

^†^ indicates diabetes, hypertension, coronary disease, kidney failure, nervous system disease, and chronic obstructive pulmonary disease. ^‡^ indicates carcinoembryonic and squamous cell carcinoma antigens.

SCC, squamous cell carcinoma; ADC, adenocarcinoma; VATS, video-assisted thoracic surgery.

### 3.2 Pathological findings

Comparisons of the pathological findings between the SCC group and the ADC group are shown in **Table 2**. Differences were observed in terms of tumor diameter (*P*=0.002), visceral pleural (*P*=0.002), Ki-67 index (*P*=0.001), pT1 stage (*P*=0.032), and station 4L metastasis (*P*=0.005). The incidence of station 4L metastasis was 17.1% (27/158) in the entire cohort, with 8.1% in the SCC group and 25.0% in the ADC group. Two patients had skip station 4L metastasis in the ADC group and one patient had solitary station 4L metastasis in the SCC group. Four patients in the ADC group only had station 4L and 10 LN metastasis.

**Table 2:** Pathological findings in the squamous cell carcinoma and adenocarcinoma groups.

Variables	SCC	ADC	*P*
	N=74	N=84	
Tumor diameter(mm)	35(25.5, 50)	30(20, 45)	0.002
Visceral pleural invasion	19(25.7%)	42(50.0%)	0.002
Lymphovascular invasion	6(8.1%)	9(10.7%)	0.577
Ki-67 index (%)	50(32.5, 75)	25(10,50)	0.001
Cell differentiation			
Well	3(4.1%)	1(1.2%)	0.341
Moderate	39(52.7%)	47(56.0%)	0.682
Poor	32(43.2%)	36(42.9%)	0.961
pT stage			
T1	15(20.3%)	30(35.7%)	0.032
T2	41(55.4%)	44(52.4%)	0.704
T3	12(16.2%)	8(9.5%)	0.207
T4	6(8.1%)	2(2.4%)	0.148
pN stage			
N0	43(58.1%)	45(53.6%)	0.567
N1	24(32.4%)	31(36.9%)	0.556
N2	14(18.9%)	28(33.3%)	0.041
Station 4L metastasis	6(8.1%)	21(25.0%)	0.005
Station 5 metastasis	8 (10.8%)	18(21.4%)	0.072
Station 6 metastasis	1(1.4%)	5(6.0%)	0.131
Station 7 metastasis	6(8.1%)	5(6.0%)	0.595
Station 8 metastasis	0	1(1.2%)	1.000
Station 9 metastasis	0	3(3.6%)	0.101
Station 10 metastasis	18(24.3%)	18(21.4%)	0.665
Station 11 metastasis	13(17.6%)	14(16.7%)	0.881
Station 12 metastasis	2(2.7%)	2(2.4%)	1.000
pTNM stage			
I	29(39.2%)	36(42.9%)	0.640
II	24(32.4%)	17(20.2%)	0.080
III	21(28.4%)	31(36.9%)	0.255
IV	0	0	1.000

SCC, squamous cell carcinoma; ADC, adenocarcinoma.

### 3.3 Risk factor analysis for 4L lymphatic metastasis

As shown in **Table 3**, the 4L metastasis was significantly correlated with abnormalities in tumor markers (*P*=0.031), histology (*P*=0.005), visceral pleural invasion (*P*<0.001), lymphovascular invasion (*P*=0.001), moderate and poor cell differentiation (*P*=0.001 and *P* < 0.001, respectively), and other stations (station 5, *P* < 0.001; station 6, *P*=0.008; station 7, *P*=0.010; station 10, *P*<0.001). These statistically significant factors were further analyzed by multivariate logistic analysis, and the results revealed that histology (SCC *vs*. ADC: OR, 0.185; 95% CI, 0.049–0.706; *P*=0.013), station 5 metastasis (OR, 20.567; 95% CI, 5.520–76.6939; *P*<0.001), station 10 metastasis (OR, 10.607; 95% CI, 2.985–37.694; *P*<0.001), and poor cell differentiation (OR, 6.080; 95% CI, 1.655–22.340; *P* =0.007) were independently associated with 4L LN metastasis.

**Table 3:** Univariate and multivariate analysis of the correlation between clinicopathological factors and station 4L metastasis.

Variables	Univariate Analysis			Multivariate Analysis		
	Station 4L Metastasis					
	positive	negative	*P*	OR	95% *CI*	*P*
Abnormality of tumor markers	8	17	0.031	0.623	0.069 to 5.650	0.674
Histology			0.005	3.778	1.432 to 0.706	0.007
SCC	6	68				
ADC	21	63				
Visceral pleural invasion	20	41	<0.001	4.284	0.573 to 32.011	0.156
Lymphovascular invasion	7	8	0.001	5.823	0.704 to 48.162	0.102
Station 5 metastasis	17	9	<0.001	20.567	5.520 to 76.6939	<0.001
Station 6 metastasis	4	2	0.008	0.297	0.013 to 6.741	0.446
Station 7 metastasis	5	6	0.010	11.479	0.812 to 162.330	0.071
Station 10 metastasis	17	19	<0.001	10.607	2.985 to 37.694	<0.001
Cell differentiation						
Well	0	4	1.000			
Moderate	7	79	0.001	0.000	NA	0.999
Poor	20	48	<0.001	6.080	1.655 to 22.340	0.007

SCC, squamous cell carcinoma; ADC, adenocarcinoma; OR, odds ratio; CI, confidence interval.

### 3.4 Survival outcomes

**Figure 2:**
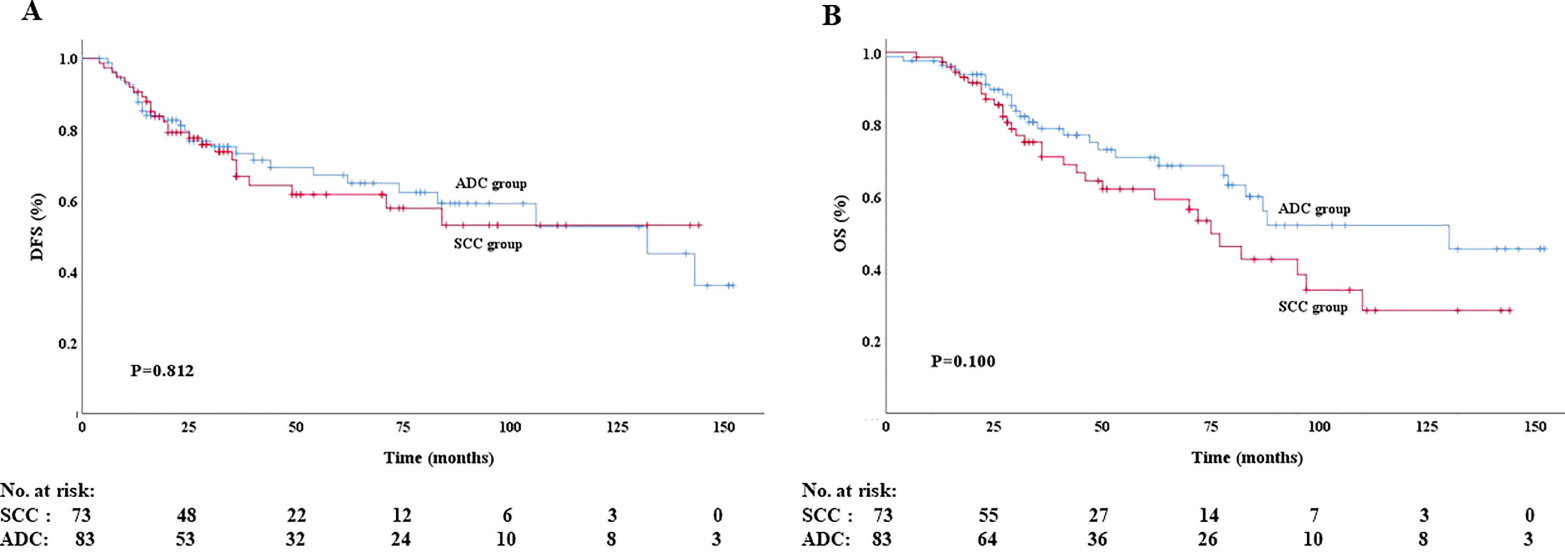
Disease-free survival **(A)** and overall survival **(B)** of patients in the ADC group and SCC group.

The median follow-up time was 36 months (range: 1–152 months). Fifty-seven patients died and 54 had recurrence or metastasis at the last follow-up. In the ADC group, 20 patients died and 20 patients had recurrence or metastasis. The rates of local recurrence and distant metastasis were 28.6% (24/84) and 23.8% (20/84), respectively. In the SCC group, 30 patients died and 34 patients had recurrence or metastasis. The rates of local recurrence and distant metastasis were 32.4% (24/74) and 16.2% (12/74), respectively. The 5-year DFS rates were 67.1% and 61.7% in the ADC group and SCC group, respectively. The 5-year OS rates in the two groups were 68.6% and 59.3%, respectively. The log-rank test showed no statistical differences in DFS (*P* =0.812; **Figure 2A**) and OS (*P* =0.100; **Figure 2B**) between the two groups.

### 3.5 Analysis of survival factors

Some variables, such as sex, station 4L metastasis, station 7 metastasis, pT stage, and pTNM stage were significantly associated with DFS in the univariate analysis (*P*=0.042, *P*<0.001, *P*<0.001, *P*<0.001, and *P*<0.001, respectively). And the above five variables were also significantly associated with OS (*P*=0.003, *P*<0.001, *P*<0.001, *P*<0.001, and *P*<0.001, respectively). Additional multivariate analysis showed that status of 4L metastasis was an independent factor for DFS (HR, 2.563; 95% CI, 1.282–5.123; *P*=0.008), together with the status of station 7 LN metastasis, pT stage, and pTNM stage. Sex, status of station 7 LN metastasis, and pTNM stage were independent factors for OS (**Table 4**).

**Table 4:** Univariate and multivariate Cox regression analysis of prognostic factors in the SCC and ADC groups.

Predictor	Univariate Analysis					Multivariate Analysis			
	DFS			OS		DFS		OS	
	*P*	95% *CI*		*P*	95% *CI*	*P*	95% *CI*	*P*	95% *CI*
Sex	0.042	2.041 (1.026 to 4.061)		0.003	3.336 (1.511 to 7.365)	0.342	1.416 (0.691 to 2.901)	0.002	3.566 (1.607 to 7.917)
Station 4L metastasis	<0.001	3.870 (2.155 to 6.947)		<0.001	3.733 (2.116 to 6.585)	0.008	2.563 (1.282 to 5.123)	0.225	1.597 (0.749 to 3.402)-
Station 7 metastasis	<0.001	8.484 (3.876 to 18.569)		<0.001	NA	<0.001	7.138 (2.991 to 17.035)	0.001	4.631 (2.032 to 10.555)
pT stage	<0.001	2.122 (1.567 to 2.874)		<0.001	1.833 (1.372 to 2.449)	0.021	2.800 (1.168 to 6.708)	0.129	1.336 (0.919 to 1.941)-
pTNM stage	<0.001	3.087 (2.121 to 4.491)		<0.001	2.764 (1.956 to 3.905)	0.007	3.911 (1.464 to 10.450)	<0.001	0.144 (0.064 to 0.323)

DFS, disease-free survival; OS, overall survival. OR, odds ratio; CI, confidence interval; SCC, squamous cell carcinoma; ADC, adenocarcinoma.

## 4 Discussion

Lymph node metastasis is a major metastatic pathway in NSCLC, which leads to poor prognosis. Thorough removal of lymph nodes is of great importance for precise stage assessment, prognosis prediction, and development of postoperative therapeutic strategies (14). The American College of Surgeons Oncology Group Z0030 trial suggested that all patients should undergo comprehensive lymph node evaluation (15). However, whether 4L LN should be resected remains unclear, as only the European Society of Thoracic Surgeons expert consensus guidelines and NCCN guidelines recommend 4L nodal evaluation for left-sided tumors, except in select circumstances (16) whereas others have no specific requirements (3, 17, 18). Wang et al. (7) raised the debate for a more comprehensive evaluation of the 4L station in patients with left-sided NSCLC.

In this study, we investigated the differences in clinicopathological features and survival outcomes between left-sided ADC and SCC after 4L LND. Our findings suggested that station 4L metastasis was not rare(17.1%), which is consistent with previous studies (7–12), and that patients with adenocarcinoma are more likely to have 4L lymph node metastasis. However, we found no difference in DFS and OS between the ADC and the SCC group. The status of station 4L metastasis was an independent factor for DFS, but not for OS.

A recent meta-analysis (12) and previous studies (7–11) had found that dissection of the 4L LN could significantly improve both the 5-year OS and DFS rates in patients with left-sided NSCLC. Specifically, Zhao et al. (9) found that patients with stage II, IIIA, and N2 disease in the 4L LND group had better survival outcomes than those without, whereas patients with stage I left-sided NSCLC had no survival benefit. Yang et al. (8) found that 4L LND only benefits patients with NSCLC in the left upper lobe, indicating that 4L LND may be unnecessary for left lower lobe tumors. From another perspective, our study found that patients with adenocarcinoma were more likely to have 4L lymph nodal metastasis than those with squamous cell carcinoma (25.0% *vs*. 8.1%; *P*=0.005), which may be the first report. We propose that it is of great necessity for left-sided adenocarcinoma to undergo 4L LND. To our knowledge, ADC develops and progresses quickly and has a poorer prognosis than SCC. However, no differences in DFS (*P*=0.812) and OS (*P*=0.100) between the ADC group and SCC group were observed in our cohort. We speculated that two main reasons may contribute to the contradictory results. First, dissection of 4L LN in the ADC group could yield more lymph nodes for examination and lead to more accurate node upstaging followed by adjuvant therapy, which might improve the DFS and OS. In our study, two patients (2.4%) had skip station 4L metastasis in the ADC group and one patient (1.4%) had single-station 4L metastasis in the SCC group, while four patients (4.8%) in the ADC group only had station 4L and 10 LN metastasis. This indicated that two patients were upstaged from N0 to N2 and four from N1 to N2 in the ADC group, and one patient was upstaged from N0 to N2 in the SCC group. Second, 4L LND could therapeutically clear lymph nodes with micrometastases, which might significantly reduce the risk of recurrence. Therefore, the poor prognosis in ADC patients may be compensated by the complete dissection of 4L LN and adjuvant treatment resulting from accurate node staging.

Fang et al. (11) demonstrated that cN2, stations 5 and 10 metastases were independent risk factors for station 4L metastasis, whereas Wang et al. (7) suggested that station 10 metastasis was independently associated with 4L metastasis. Our study revealed that histology, station 5 metastasis, station 10 metastasis, and poor cell differentiation were risk factors, implying that stations 5 and 10 metastases were common risk factors for station 4L metastasis. We speculate that this may be related to their transition zone (such as the aortopulmonary window and tracheobronchial angle) (19), which could explain the result that left upper lobe tumors had a greater preference for superior mediastinal LN metastasis than lower lobe tumors (20) and that 4L LND only benefits patients with NSCLC in the left upper lobe (8).

Interestingly, our study suggested that the status of station 4L metastasis is an independent factor for DFS, but not for OS, while the status of station 7 metastasis and pTNM stage were both independent predictors for DFS and OS. We speculate that the following reason may contribute to this finding. Station 4L LN involvement changed the pathological stage and remodeled the postoperative regimens, which temporarily influenced the DFS. However, overall survival still needs to be elevated in locally advanced and metastatic NSCLC, even with the rapid progress in immunotherapy and targeted therapy (21, 22).

With the technical development of video-assisted thoracic surgery, the safety and thoroughness of 4L LND have been demonstrated in several studies (23, 24). The rate of hoarseness (5.7%, 9/158) caused by left recurrent laryngeal nerve injury in our study was acceptable and consistent with that in previous studies (9). Therefore, concern regarding hoarseness as a complication is not an obstacle in 4L LND, and the survival benefit should be taken into consideration and consensus regarding 4L LND should be reached.

Our study has several limitations. First, this single-center retrospective study inevitably had the possibility of uncontrolled confounding or selection bias, and we could not use the propensity score matching method to reduce them because of the relatively small number of enrolled patients with 4L LND left-sided tumors. This could be overcome by using a larger sample size and conducting a multicenter randomized clinical trial. Second, we focused on patients who received 4L LND without comparing them to patients who did not undergo 4L LND, as in some other studies. These findings should not be overinterpreted.

In conclusion, station 4L metastasis is not rare in left lung cancer. Patients with adenocarcinoma have a greater predilection for station 4L metastasis and may benefit more from performing 4L LND.

## Data availability statement

The raw data supporting the conclusions of this article will be made available by the authors, without undue reservation.

## Ethics statement

The studies involving human participants were reviewed and approved by Ethics Committee of Chinese People’s Liberation Army General Hospital. The ethics committee waived the requirement of written informed consent for participation.

## Author contributions

LS: Conceptualization, Methodology, Data curation, Writing-Original draft preparation. JG: Data curation, Writing-Review and Editing. HC: Formal analysis. WZ: Conceptualization, Writing-Review and Editing. YL: Conceptualization, Supervision, Writing-Reviewing and Editing. All authors contributed to the article and approved the submitted version.
